# Exploring the multiple-hit hypothesis of preterm white matter damage using diffusion MRI

**DOI:** 10.1016/j.nicl.2017.11.017

**Published:** 2017-11-21

**Authors:** Madeleine L. Barnett, Nora Tusor, Gareth Ball, Andrew Chew, Shona Falconer, Paul Aljabar, Jessica A. Kimpton, Nigel Kennea, Mary Rutherford, A. David Edwards, Serena J. Counsell

**Affiliations:** a Centre for the Developing Brain, Division of Imaging Sciences & Biomedical Engineering, King's College London, London SE1 7EH, UK; b St George's Hospital NHS Trust, Blackshaw Road, London SW17 0QT, UK

**Keywords:** ALIC, anterior limb of the internal capsule, BSITD-III, Bayley Scales of Infant and Toddler Development Third Edition, dMRI, diffusion magnetic resonance imaging, GA, gestational age, IMD, index of multiple deprivation, PMA, postmenstrual age, PLIC, posterior limb of the internal capsule, SLF, superior longitudinal fasciculus, Brain, Development, Diffusion MRI, Prematurity, Multiple hit hypothesis

## Abstract

**Background:**

Preterm infants are at high risk of diffuse white matter injury and adverse neurodevelopmental outcome. The multiple hit hypothesis suggests that the risk of white matter injury increases with cumulative exposure to multiple perinatal risk factors. Our aim was to test this hypothesis in a large cohort of preterm infants using diffusion weighted magnetic resonance imaging (dMRI).

**Methods:**

We studied 491 infants (52% male) without focal destructive brain lesions born at < 34 weeks, who underwent structural and dMRI at a specialist Neonatal Imaging Centre. The median (range) gestational age (GA) at birth was 30^+ 1^ (23^+ 2^–33^+ 5^) weeks and median postmenstrual age at scan was 42^+ 1^ (38–45) weeks. dMRI data were analyzed using tract based spatial statistics and the relationship between dMRI measures in white matter and individual perinatal risk factors was assessed. We tested the hypothesis that increased exposure to perinatal risk factors was associated with lower fractional anisotropy (FA), and higher radial, axial and mean diffusivity (RD, AD, MD) in white matter. Neurodevelopmental performance was investigated using the Bayley Scales of Infant and Toddler Development, Third Edition (BSITD-III) in a subset of 381 infants at 20 months corrected age. We tested the hypothesis that lower FA and higher RD, AD and MD in white matter were associated with poorer neurodevelopmental performance.

**Results:**

Identified risk factors for diffuse white matter injury were lower GA at birth, fetal growth restriction, increased number of days requiring ventilation and parenteral nutrition, necrotizing enterocolitis and male sex. Clinical chorioamnionitis and patent ductus arteriosus were not associated with white matter injury. Multivariate analysis demonstrated that fetal growth restriction, increased number of days requiring ventilation and parenteral nutrition were independently associated with lower FA values. Exposure to cumulative risk factors was associated with reduced white matter FA and FA values at term equivalent age were associated with subsequent neurodevelopmental performance.

**Conclusion:**

This study suggests multiple perinatal risk factors have an independent association with diffuse white matter injury at term equivalent age and exposure to multiple perinatal risk factors exacerbates dMRI defined, clinically significant white matter injury. Our findings support the multiple hit hypothesis for preterm white matter injury.

## Highlights

• White matter injury was assessed in 491 preterm infants at term equivalent age.
• Aberrant white matter development was associated with several perinatal factors.
• Our findings support the multiple hit hypothesis for preterm brain injury.

## 1 Introduction

Preterm birth is a leading cause of perinatal mortality and morbidity, and creates significant personal, social and healthcare costs. Around 15 million infants are born preterm each year, and this incidence is increasing (WHO, 2013). While survival rates following preterm birth have improved, the incidence of severe neurodevelopmental deficits remains unchanged (Moore et al., 2012). The developing white matter is particularly susceptible to injury (Volpe, 2009). Diffusion weighted magnetic resonance imaging (dMRI) allows detailed assessment of white matter injury in the neonatal period and has identified altered white matter development following preterm birth. Compared to their term-born peers, preterm infants display significantly lower fractional anisotropy (FA) throughout the white matter on dMRI (Anjari et al., 2007, Counsell et al., 2006, Hüppi et al., 1998, Rose et al., 2008). These diffusion changes are part of more global differences in brain development following premature delivery (Ajayi-Obe et al., 2000, Ball et al., 2012, Ball et al., 2013a, Boardman et al., 2006, Dubois et al., 2008, Kersbergen et al., 2016) which relate to neurodevelopmental performance (Ball et al., 2015, Counsell et al., 2008, Van Kooij et al., 2012, Thompson et al., 2014, Duerden et al., 2015, Guo et al., 2017).

Animal models provide evidence that preterm white matter injury may be related to more than one risk factor (Ådén et al., 2010, Campbell et al., 2012, Penn et al., 2016, Van Steenwinckel et al., 2014). These findings have led to the multiple hit hypothesis of preterm brain injury, where antenatal factors may sensitize brain tissue, rendering it vulnerable to injury from secondary events in the early postnatal period (Van Steenwinckel et al., 2014).

This hypothesis is supported in part by neuroimaging studies in human preterm infants which have shown that preterm white matter injury is related to multiple factors including low gestational age (GA), respiratory disease, male sex, poor nutrition, infection, chorioamnionitis, pain, stress and illness severity (Ball et al., 2010, Counsell et al., 2008, Kuypers et al., 2012, Rose et al., 2009, Tan et al., 2008, Anblagan et al., 2016, Smith et al., 2011, Chau et al., 2012, Brummelte et al., 2012, Zwicker et al., 2013, Beauport et al., 2017). However, these factors all have high degrees of co-linearity, and it is not clear if they represent a single latent variable which can be largely captured by inclusion of any one in an analysis, or whether each carries a separate risk which is additive to other risk factors. This study aimed to address this issue by testing the hypothesis that increased exposure to perinatal risk factors was associated with lower FA and higher radial, axial and mean diffusivity (RD, AD, MD) in white matter. We also tested the hypothesis that lower FA and higher RD, AD and MD in white matter at term equivalent age were associated with poorer neurodevelopmental performance in early childhood.

## 2 Methods

The National Research Ethics Service and the Hammersmith, Queen Charlotte's and Chelsea Hospital Research Ethics Committees granted ethical permission for MRI (09/H0707/98, 04/Q0406/125, 06/Q0406/14 07/H070/101). Written parental consent was obtained prior to imaging.

### 2.1 Subjects

We studied the imaging and clinical data from 614 preterm infants, recruited as part of the Evaluation of Preterm Imaging Study (ePrime) (NCT01049594) study from hospitals within North and South West London Perinatal Network, and from on-going imaging studies within the Neonatal Intensive Care Unit at Queen Charlotte's and Hammersmith Hospitals from June 2006 to November 2012. Infants born at < 34 weeks GA and scanned at term equivalent age (between 38 and 45 weeks postmenstrual age, PMA) without major congenital malformation or metal implants were eligible for inclusion in the study.

Infants with major focal lesions such as periventricular leukomalacia, hemorrhagic parenchymal infarction and other ischemic or hemorrhagic lesions (*n* = 75) were excluded from analysis (Table S1). 48 subjects were excluded due to motion corrupt dMRI (> 8 dMRI volumes). The final study population consisted of 491 preterm infants (256 males) who were born at a median GA of 30^+ 1^ (range 23^+ 2^–33^+ 5^) weeks and PMA at scan of 42^+ 1^ (range 38–45) weeks. The perinatal characteristics of these infants are presented in Table 1.

**Table 1:** Demographic, clinical characteristics and neurodevelopmental outcome of infants.

Perinatal characteristic	Available for analysis (n)	
GA at birth, median (range) in weeks	491	30^+ 1^ (23^+ 2^–33^+ 5^)
PMA at scan, median (range) in weeks	491	42^+ 1^ (38^+ 0^–45^+ 0^)
Birth weight, median (range) in grams	490	1260 (350–2600)
Males, no (%)	491	256 (52.1)
Birth set, singletons/twins, no (%)	491	347(70.7)/144 (29.3)
Days on mechanical ventilation, median (range) in days	467	0 (0–40)
Days on parenteral nutrition, median (range) in days	386	6 (0–89)
Chorioamnionitis, no (%)	461	33 (7.2)
Fetal growth restriction, no (%)	463	80 (17.3)
Necrotizing enterocolitis requiring surgery, no (%)	471	7 (1.5)
Patent ductus arteriosus requiring medical or surgical management, no (%)	468	22 (4.7)
Index of multiple deprivation, mean (range)	348	18.5 (1.7–61.3)

Neurodevelopmental assessment		
Cognitive score mean (sd) Cognitive score < 85 Cognitive score < 70	381 62 (16.3%) 9 (2.4%)	94 (13)
Motor score mean (sd) Motor score < 85 Motor score < 70	380 32 (8.4%) 6 (1.6%)	97 (11)
Language score mean (sd) Language score < 85 Language score < 70	380 117 (30.8%) 37 (9.7%)	92 (17)

Abbreviations: GA, gestational age; PMA, postmenstrual age.

### 2.2 Demographic and clinical data collection

Demographic and clinical data were collected from the Standardized Electronic Neonatal Database (SEND). Variables of interest included GA at birth, PMA at scan, gender, days spent on invasive ventilation via an endotracheal tube (up to the date of MRI), days of parenteral nutrition (up to the date of MRI), the presence of chorioamnionitis (determined by the obstetric team, rather than a histological diagnosis), the presence of fetal growth restriction (diagnosed by the obstetric team at the hospital where antenatal care was provided), necrotizing enterocolitis requiring surgical intervention, the presence of a patent ductus arteriosus requiring medical or surgical treatment, and Index of Multiple Deprivation (IMD) score (a marker of socio-economic status based on seven domains of deprivation; Income Deprivation, Employment Deprivation, Education, Skills and Training Deprivation, Health Deprivation and Disability, Crime, Barriers to Housing and Services and Living Environment Deprivation), determined by the postcode of the parent at the time of infant birth (http://imd-by-postcode.opendatacommunities.org).

### 2.3 MR imaging

3D MPRAGE (Magnetization Prepared Rapid Acquisition Gradient Echo, TR 17 ms; TE 4.6 ms; flip angle 13°; slice thickness 0.8 mm; in plane resolution 0.82 × 0.82 mm), T2-weighted turbo spin echo (TR 8670 ms; TE 160 ms; flip angle 90°; slice thickness 2 mm; in plane resolution 0.86 × 0.86 mm) and single shot echo planar dMRI (TR 7536 ms; TE 49 ms; flip angle 90°; slice thickness 2 mm; in plane resolution 2 × 2 mm, 32 non-collinear gradient directions, *b* value of 750 s/mm^2^) were acquired on a Philips 3 Tesla (Philips Medical Systems, Best, The Netherlands) system sited on the neonatal intensive care unit using an eight-channel phased array head coil.

All examinations were supervised by a pediatrician experienced in MR imaging. Parents were offered sedation for their child, oral chloral hydrate (25–50 mg/kg), prior to scanning and 398 infants (81%) were sedated for imaging. Pulse oximetry, temperature and electrocardiography were monitored throughout the scan and ear protection was used, comprising earplugs molded from a silicone-based putty (President Putty, Coltene Whaledent, Mahwah, NJ, USA) placed in the external auditory meatus and neonatal earmuffs (MiniMuffs, Natus Medical Inc., San Carlos, CA, USA).

### 2.4 Image processing

dMRI images were assessed visually for the presence of motion artifact and if ≤ 8 volumes were corrupt, these volumes were removed. If > 8 dMRI volumes showed motion artifact, they were excluded from analysis. 143/491 datasets had at least one volume removed (median 1, range 0–8). Image processing and data analysis were performed using FMRIB's Diffusion Toolbox (v3.0), DTI-ToolKit (v2.3.1 www.dti-tk.sourceforge.net) (DTI-TK) (Zhang et al., 2006) and tract based spatial statistics (TBSS) (v1.2 http://fsl.fmrib.ox.ac.uk/fsl/fslwiki/TBSS/UserGuide) (Smith et al., 2006) as implemented in FMRIB's Software Library (FSL v5.0; www.fmrib.ox.ac.uk/fsl). For each infant the diffusion weighted images were registered to their native b0 image and corrected for differences in spatial distortion using eddy correct. Non-brain tissue was removed with FSL's Brain Extraction Tool (BET v2.1 http://fsl.fmrib.ox.ac.uk/fsl/fslwiki/BET).

Diffusion tensors were calculated on a per voxel basis, using a simple least squares fit of the tensor model to the diffusion data. From this the tensor eigenvalues describing the diffusion strength in the primary, secondary and tertiary diffusion directions were obtained. Axial diffusivity (AD), radial diffusivity (RD), mean diffusivity (MD) and FA maps were calculated for each subject.

Image registration was performed using DTI-TK and integrated within the TBSS pipeline to produce a population specific DTI template. From this template a mean FA map was derived and then thinned by perpendicular non-maximum suppression to create a mean FA skeleton. A FA threshold of ≥ 0.15 was used to limit the inclusion of voxels with high inter-subject variability and non-white matter voxels. FA, AD, RD and MD were projected onto this skeleton prior to statistical analysis.

### 2.5 Neurodevelopmental outcomes

Of 491 subjects who had suitable dMRI data 381 (77.6%) returned for neurodevelopmental assessment at a median of 20.23 months corrected age (Table 1). Neurodevelopmental performance was assessed using the Bayley Scales of Infant and Toddler Development, Third Edition (BSITD-III; Bayley, 2006) and cognitive, language and motor composite scores were obtained. One child failed to complete the motor component and another child failed to complete the language component of the test.

### 2.6 Statistical analysis

#### 2.6.1 Relationship between dMRI measures and individual perinatal risk factors

In order to investigate the relationship between dMRI metrics in white matter at term equivalent age and clinical risk factors, cross-subject voxelwise statistical analysis was performed using Randomise in FSL (v2.9) (http://fsl.fmrib.ox.ac.uk/fsl/fslwiki/Randomise). A general linear model (GLM) was used to assess the relationship between dMRI metrics and GA at birth, PMA at scan and gender were included as covariates in the model. Differences between male and female infants were assessed using a GLM, with GA at birth and PMA at scan as co-variates. GA at birth, PMA at scan and gender were included as covariates in all subsequent analyses of perinatal risk factors; number of days requiring mechanical ventilation, number of days requiring parenteral nutrition, chorioamnionitis, fetal growth restriction, necrotizing enterocolitis requiring surgery, patent ductus arteriosus requiring medical or surgical treatment (primary outcome measure: FA; secondary outcome measures; AD, MD and RD; predictors: perinatal risk factors, PMA at scan).

#### 2.6.2 Multivariate analysis of the relationship between dMRI measures and perinatal risk factors

We used a multivariable model, which included those perinatal risk factors that demonstrated a significant relationship with dMRI measures on analysis of individual risk factors. We included fetal growth restriction, necrotizing enterocolitis requiring surgery, days requiring invasive ventilatory support and days requiring parenteral nutrition, with GA at birth, PMA at scan and gender included as covariates in the model.

#### 2.6.3 Relationship between dMRI measures and cumulative perinatal risk factor score

In order to assess the relationship between exposure to multiple clinical risk factors and white matter injury we formulated a score of cumulative risk factors for each infant. A binary scoring system was devised as follows; fetal growth restriction or necrotizing enterocolitis requiring surgery were scored as 1, invasive ventilatory support ≥ 7 days was scored as 1, and parenteral nutrition ≥ 5 days was scored as 1. These scores were added together to give a minimum possible score of 0 and maximum possible score of 4, and GLM analysis performed in TBSS with GA at birth, PMA at scan and gender as covariates in the model.

#### 2.6.4 Relationship between dMRI measures at term equivalent age and subsequent neurodevelopmental performance

A GLM was used to assess the relationship between dMRI metrics in white matter at term equivalent age and cognitive, motor and language performance at 2 years, with GA at birth, PMA at scan, gender and IMD score included as co-variates (outcome: FA, AD, MD and RD measures; predictors: cognitive, motor and language performance, GA at birth, PMA at scan, gender and IMD score). All statistical analyses were subject to family-wise error (FWE) correction for multiple comparisons following threshold-free cluster enhancement (TFCE) and p < 0.05 was considered significant.

## 3 Results

### 3.1 Demographic data

The perinatal clinical characteristics of the infants are shown in Table 1.

### 3.2 Relationship between dMRI measures in white matter and perinatal risk factors

#### 3.2.1 Gestational age at birth

**Fig. 1:**
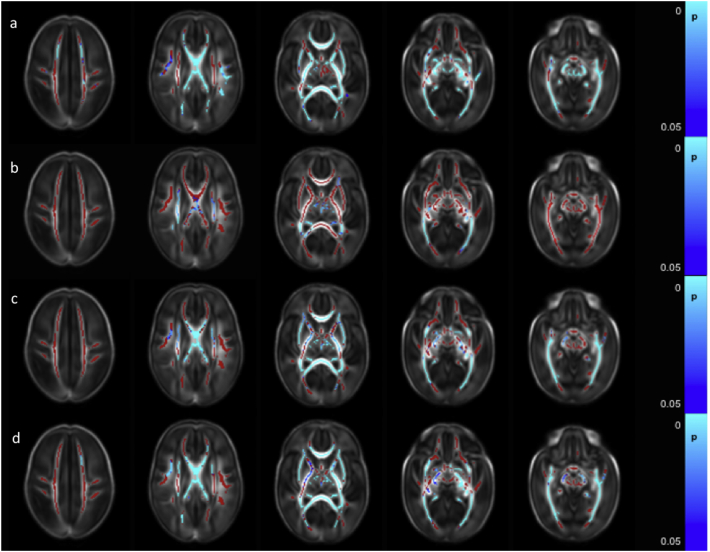
Correlation between gestational age at birth and dMRI measures in white matter. Mean FA skeleton (red) overlaid on mean FA map in the axial plane. Voxels showing a significant correlation (p < 0.05) between GA at birth and a. FA, b. AD c. MD, and d. RD are shown in blue-light blue. (For interpretation of the references to color in this figure legend, the reader is referred to the web version of this article.)

FA values were positively correlated with GA at birth throughout the white matter (Fig. 1a and Fig. s1). AD was negatively correlated with GA at birth in the anterior limb of the internal capsule (ALIC) bilaterally, posterior limb of the internal capsule (PLIC) bilaterally, corpus callosum, bilateral fornix, optic radiation and inferior longitudinal fasciculus/inferior fronto-occipital fasciculus (ILF/IFOF) (Fig. 1b) and positively correlated in the centrum semi-ovale (not shown). MD was negatively correlated with GA at birth in the corpus callosum, bilateral fornix, external capsule, ALIC, PLIC, optic radiation, ILF/IFOF bilaterally and the right crus cerebri (Fig. 1c). RD was negatively correlated with GA at birth in the left frontal white matter, bilateral cingulum, corpus callosum, external capsule, ALIC, PLIC, optic radiation, ILF/IFOF and the crus cerebri bilaterally (Fig. 1d).

#### 3.2.2 Sex

**Fig. 2:**
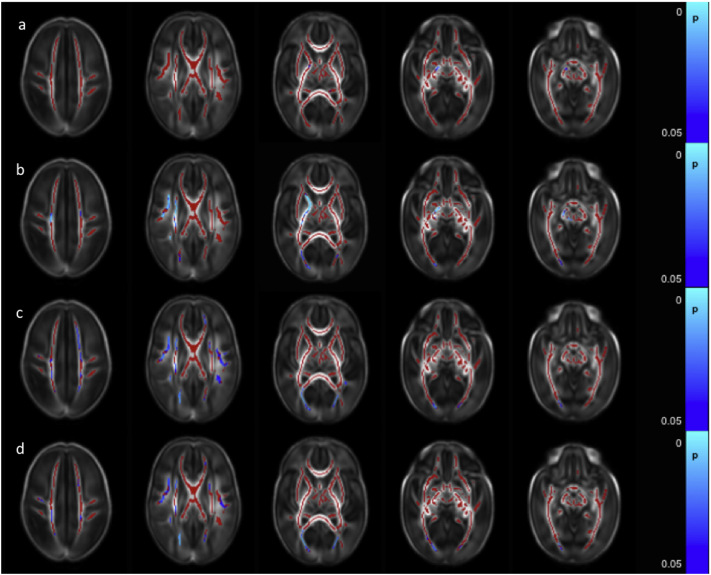
Difference in white matter dMRI measures between male and female infants. Mean FA skeleton (red) overlaid on mean FA map in the axial plane. Voxels showing significantly greater (p < 0.05) a. FA, b. AD c. MD, and d. RD in male infants compared to female infants are shown in blue-light blue. (For interpretation of the references to color in this figure legend, the reader is referred to the web version of this article.)

FA values were higher in only a very few voxels in the right PLIC and the right crus cerebri in male infants (Fig. 2a). AD was higher in male infants in the right superior longitudinal fasciculus (SLF), centrum semiovale bilaterally, right ALIC, bilateral PLIC, right optic radiation and right crus cerebri (Fig. 2b). MD values were higher in the male infants in the centrum semiovale bilaterally, left frontal white matter, SLF, ALIC, PLIC, and optic radiation bilaterally (Fig. 2c). RD was higher in the male infants in the right PLIC and the centrum semiovale, SLF, ALIC and optic radiation bilaterally (Fig. 2d).

#### 3.2.3 Chorioamnionitis

There were no significant differences in dMRI measures between those infants who had a clinical diagnosis of chorioamnionitis and those who did not.

#### 3.2.4 Fetal growth restriction

**Fig. 3:**
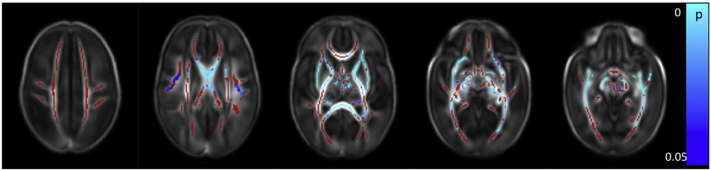
Lower FA values in the white matter in infants with fetal growth restriction. Mean FA skeleton (red) overlaid on mean FA map in axial plane. Voxels showing significant lower (p < 0.05) FA values in the white matter in infants with fetal growth restriction are shown in blue-light blue. (For interpretation of the references to color in this figure legend, the reader is referred to the web version of this article.)

FA values were significantly lower in infants with fetal growth restriction in the corpus callosum, right cingulum and SLF, external capsule, ALIC, PLIC, fornix, optic radiation, ILF/IFOF, crus cerebri and cerebellar peduncles bilaterally (Fig. 3). No significant differences between infants with fetal growth restriction and appropriately grown infants were observed in AD, MD or RD values.

#### 3.2.5 Days on mechanical ventilation

**Fig. 4:**
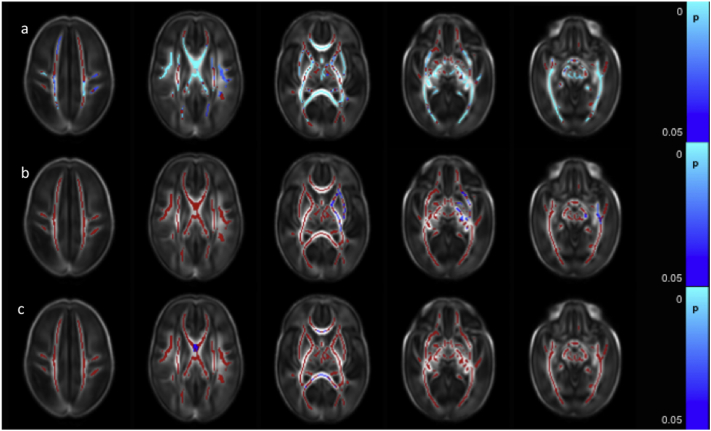
Correlation between days requiring invasive ventilation and dMRI measures in white matter. Mean FA skeleton (red) overlaid on mean FA map in axial plane. Voxels showing a significant correlation (p < 0.05) between days of ventilation and a. FA, b. AD, and c. RD are shown in blue-light blue. (For interpretation of the references to color in this figure legend, the reader is referred to the web version of this article.)

FA values throughout the white matter were negatively correlated with the number of days on mechanical ventilation (Fig. 4a and Fig. s2). AD values were negatively correlated with the number of days on mechanical ventilation in the left ALIC, left PLIC, left external capsule, left crus cerebri and in the left anterior ILF (Fig. 4b). RD values were positively correlated with the number of days on mechanical ventilation in the corpus callosum (Fig. 4c). There were no significant correlations between the number of days on mechanical ventilation and MD values.

#### 3.2.6 Days on parenteral nutrition

**Fig. 5:**
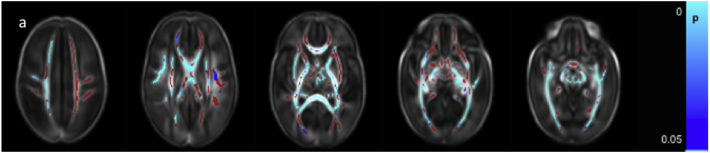
Correlation between days requiring parenteral nutrition and dMRI measures in white matter. Mean FA skeleton (red) overlaid on mean FA map in the axial plane. Voxels showing a significant correlation (p < 0.05) between FA and days of parenteral nutrition with are shown in blue-light blue. (For interpretation of the references to color in this figure legend, the reader is referred to the web version of this article.)

With the exception of the left centrum semiovale and the left external capsule, which showed no correlations with days on parenteral nutrition, FA values throughout the white matter were significantly negatively correlated with the number of days an infant received parenteral nutrition (Fig. 5 and Fig. s3). No significant correlations between days receiving parenteral nutrition and AD, MD or RD were identified.

#### 3.2.7 Necrotizing enterocolitis

**Fig. 6:**
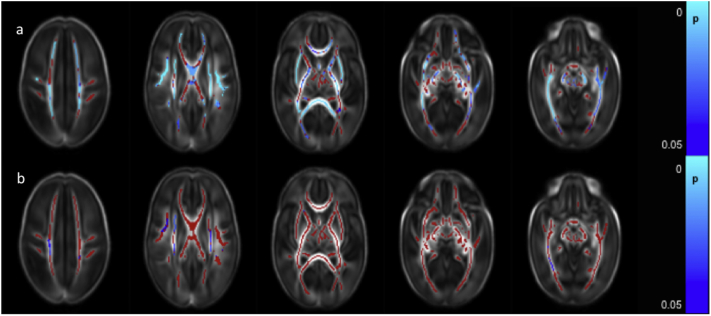
Difference between dMRI measures in the white matter in infants who had or had not undergone surgery for necrotising enterocolitis. Mean FA skeleton (red) overlaid on mean FA map in axial plane. Voxels showing a significant difference (p < 0.05) between infants with and without necrotising enterocolitis in a. FA and b. RD are shown in blue-light blue. (For interpretation of the references to color in this figure legend, the reader is referred to the web version of this article.)

FA values throughout the white matter were significantly lower in those infants who had undergone surgical treatment for necrotizing enterocolitis (Fig. 6a). RD values were higher in a small number of voxels within the centrum semiovale bilaterally, right SLF, a small region of the right ALIC and the right ILF/IFOF (Fig. 6b). There were no differences in MD and AD values between those infants who had undergone surgery for necrotizing enterocolitis and those who had not.

#### 3.2.8 Patent ductus arteriosus

There were no significant differences in dMRI measures between those infants who had undergone treatment for patent ductus arteriosus and those who had not.

### 3.3 Multivariate analysis assessing the relationship between dMRI measures and clinical risk factors

Clinical data for all risk factors were available for 381 infants, allowing multivariate analysis of all risk factors simultaneously. There were no significant differences in GA at birth, PMA at scan, gender distribution or the proportion of infants who were singletons/multiple births between the 381 infants included in the multivariate analysis and the 110 infants who were not included (Table S2). Multivariate analysis of the relationship between dMRI measures and risk factors (fetal growth restriction, necrotizing enterocolitis requiring surgery, days requiring invasive ventilatory support and days requiring parenteral nutrition) demonstrated that the extent of the relationship between risk factor and dMRI measures was diminished compared to assessing each risk factor individually. However, significant relationships between fetal growth restriction, days requiring invasive ventilatory support and days requiring parenteral nutrition and FA values remained.

#### 3.3.1 Fetal growth restriction

Multivariate analysis showed that FA values in the corpus callosum, ALIC, PLIC, external capsule crus cerebri and cerebellar peduncles were significantly lower in infants with fetal growth restriction than appropriately grown infants (Fig. s4). No significant differences between infants with fetal growth restriction and appropriately grown infants were observed in AD, MD or RD values.

#### 3.3.2 Days on mechanical ventilation

FA values in the corpus callosum, cingulum and optic radiation were negatively correlated with the number of days on mechanical ventilation (Fig. s5). There were no significant correlations between the number of days on mechanical ventilation and AD, MD or RD values.

#### 3.3.3 Days on parenteral nutrition

FA values in the corpus callosum, cingulum, right SLF, PLIC, left ALIC, optic radiation, crus cerebri and cerebellar peduncles were significantly negatively correlated with the number of days an infant received parenteral nutrition (Fig. s6). No significant correlations between days receiving parenteral nutrition and AD, MD or RD were identified.

#### 3.3.4 Necrotizing enterocolitis

There were no differences in FA, AD, MD or RD values between those infants who had undergone surgery for necrotizing enterocolitis (*n* = 7) and those who had not when assessed using multivariate analysis including all clinical risk factors.

### 3.4 Relationship between dMRI measures and cumulative perinatal risk factor score

Clinical data for all risk factors were available for 381 infants, allowing the calculation of the cumulative perinatal risk factor. Within this group, there were 149 infants with a score of 0, 183 with a score of 1, 45 with a score of 2, and 4 with a score of 3. No infant scored the maximum possible score of 4.

**Fig. 7:**
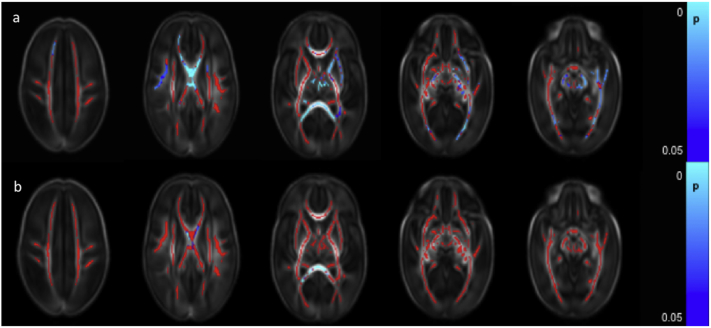
Relationship between cumulative risk factor score and dMRI white matter measures. Mean FA skeleton (red) overlaid on mean FA map in the axial plane. Voxels showing a significant linear correlation (p < 0.05) between cumulative perinatal risk factor score and a. FA and b. RD are shown in blue-light blue. (For interpretation of the references to color in this figure legend, the reader is referred to the web version of this article.)

A higher cumulative risk factor score was associated with lower FA values in the corpus callosum, left frontal white matter, left SLF, left cingulum, left ALIC, left PLIC and left external capsule, the bilateral fornix, optic radiation and the ILF/IFOF, the crus cerebri and cerebellar peduncles bilaterally (Fig. 7a and Fig. s7). RD values were positively correlated with risk factor score in the corpus callosum and the left fornix (Fig. 7b). There were no significant correlations between AD and MD and the cumulative risk factor score.

### 3.5 Relationship between dMRI measures and neurodevelopmental performance

#### 3.5.1 Cognitive performance

**Fig. 8:**
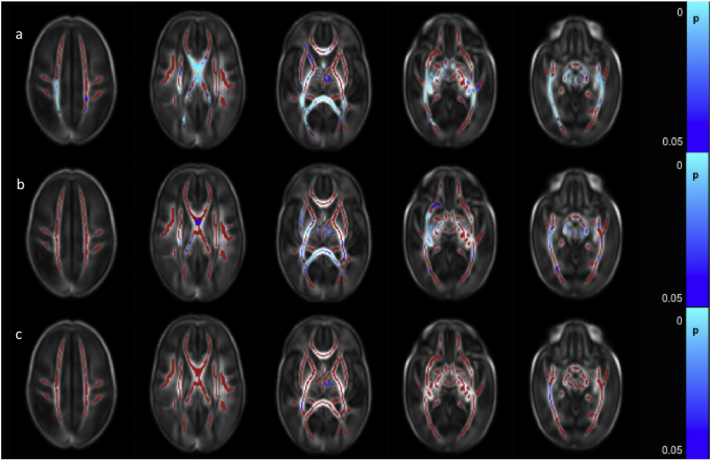
Correlation between neurodevelopmental assessment scores and FA values in the white matter. Mean FA skeleton (red) overlaid on mean FA map in the axial plane. Voxels showing a significant correlation (p < 0.05) between FA and a. Cognitive Score, b. Motor Score and c. Language Score are shown in blue-light blue. (For interpretation of the references to color in this figure legend, the reader is referred to the web version of this article.)

FA values throughout the white matter were positively correlated with composite cognitive scores from the BSITD-III. No significant association was seen between cognitive performance and AD, MD or RD (Fig. 8a).

#### 3.5.2 Motor performance

FA values in the corpus callosum, the right ALIC, the right external capsule, the PLIC, fornix, optic radiation, ILF/IFOF, crus cerebri and the cerebellar peduncles bilaterally were positively correlated with the composite score for motor function (Fig. 8b). RD was negatively correlated with motor function scores in the corpus callosum, left fornix and small areas in the optic radiation bilaterally (not shown). Neither AD nor MD demonstrated a significant association with motor score.

#### 3.5.3 Language

Language composite scores were positively correlated with FA in small regions in the body of the corpus callosum, the left fornix and the anterior aspect of right ILF/IFOF (Fig. 8c). No significant associations were seen in the other diffusion metrics.

## 4 Discussion

In this study we assessed a large cohort of preterm infants and demonstrated that preterm white matter injury is associated with multiple perinatal risk factors including immaturity at birth, fetal growth restriction, duration of mechanical ventilation, the number of days requiring parenteral nutrition, surgery for necrotizing enterocolitis and male gender. It is possible that these risk factors represent a single latent variable or marker of infant health, and if so, we may expect that exposure to additional risk factors would not result in an increased risk of white matter injury. However, multivariate analysis demonstrated lower FA values in infants with fetal growth restriction, and infants who required prolonged mechanical ventilation or total parenteral nutrition when these factors were assessed simultaneously. Furthermore, lower FA and higher RD values were observed in those infants who were exposed to multiple risk factors, assessed by our cumulative risk score, after correcting for the effects of prematurity. These data suggest that, while individual risk factors are associated with altered dMRI characteristics in the white matter, exposure to cumulative risk factors is associated with an increased vulnerability for white matter injury. These findings are consistent with recent animal work demonstrating that preterm brain injury is related to more than one risk factor and is exacerbated by exposure to multiple factors (Ådén et al., 2010, Campbell et al., 2012, Van Steenwinckel et al., 2014). Our findings thus support the multiple hit hypothesis of preterm brain injury (Dammann and Leviton, 2004, Van Steenwinckel et al., 2014).

TBSS is limited with regard to anatomical specificity, due to registration errors, use of statistical cut-offs to define significant regions which are affected by sample size and difficulties related to topological variability between adjacent and differently oriented fibres with similar FA values. We employed a tensor-based group-wise registration tool, with a study specific registration template, to produce a group specific template and skeleton which has been shown to limit these problems (Bach et al., 2014). The anatomical location of the differences described is therefore somewhat provisory, although this does not affect the overall conclusions of the study. A limitation of our study is that only linear associations were examined, and so important non-linear associations may exist and are not reported here. Furthermore, our cumulative risk score imposed equal weights for the clinical risk factors and we did not assess different weighting schemes. It is possible that the results may vary if a different weighting scheme is used. Nevertheless, our multivariate analysis shows that key risk factors, fetal growth restriction and requirement for prolonged mechanical ventilation or total parenteral nutrition, are independent risk factors for diffuse white matter injury in preterm infants.

FA values increase and MD, RD, and to a more limited extent, AD values decrease with maturation in the age-range studied here (Ball et al., 2010, Rose et al., 2014). The relationship between tissue microstructure, diffusivity and anisotropic diffusion is complex. Experimentally, anisotropy is predominantly dependent on the packing of parallel axons, axonal thickness and myelination (Sakuma et al., 1991, Takagi et al., 2009). However the preterm brain at term equivalent age is largely unmyelinated (Yakolev and Lecours, 1967, Wimberger et al., 1995) and so diminished FA and increased diffusivity is likely to involve a combination of elevated brain water content, decreases in axon diameter, increased membrane permeability, and impaired oligodendrocyte proliferation and maturation, resulting in less coherent axonal organization (Wimberger et al., 1995, Beaulieu, 2002).

Axial diffusivity represents the estimated magnitude of diffusion parallel to the direction of fibres, and RD provides an estimate of the magnitude of diffusion perpendicular to the direction of fibres. We observed a stronger relationship between elevated RD and perinatal risk factors than those between AD and perinatal risk factors, consistent with previous findings (Anjari et al., 2007, Chau et al., 2013), suggesting that decreases in FA are largely driven by increased diffusivity perpendicular to axons. However, in the absence of histology, we are limited in the conclusions we can draw with respect to the underlying microstructure. The diffusion tensor can model only a single fibre population and the presence of multiple fibre populations within a single white matter voxel (Jeurissen et al., 2013) confounds biophysical interpretations of diffusion tensor measures (Wheeler-Kingshott and Cercignani, 2009).

Diffuse white matter injury in the absence of major lesions is associated with degree of prematurity at birth (Anjari et al., 2007) and is accompanied by impaired cortical folding (Ajayi-Obe et al., 2000, Dubois et al., 2008), altered cortical microstructure (Ball et al., 2013b) and deep gray matter development (Ball et al., 2012, Boardman et al., 2006). In addition, male survivors of preterm birth have lower neurodevelopmental outcome scores and are at higher risk of cerebral palsy than preterm females (Jarvis et al., 2005). MRI studies have shown reduced gray matter to white matter ratio (Allen et al., 2003), decreased cortical thickness in adults (Luders et al., 2006) and lower FA in the splenium and the right PLIC (Rose et al., 2009) in males at term equivalent age. In this study, we identified higher AD, MD and RD in the corticospinal tracts, corpus callosum and association tracts compared to a very limited area of higher FA in the male infants. This elevated diffusivity suggests increased vulnerability to white matter injury in male infants.

Infection and inflammation are thought to increase susceptibility of the brain to injury (Coumans et al., 2003, Eklind et al., 2001, Yang et al., 2004) and is associated with diminished white matter FA values in preterm infants (Chau et al., 2012). However, obtaining reliable clinical data on sepsis is challenging. Small and often inadequate blood volumes are used for blood culture in neonates (Connell et al., 2007) and rates of false positives are high (Segal and Chamberlain, 2000). C-reactive protein is also not specific for bacterial infection (Volanakis, 2001). We did not, therefore, investigate the relationship between blood cultures and white matter injury, which is a limitation of our study. We did, however, investigate whether chorioamnionitis was related to white matter injury at term equivalent age and, consistent with Chau et al. (2009), we found that clinically defined chorioamnionitis was not associated with significant changes in dMRI measures. Not all clinical chorioamnionitis will have intra-amniotic bacteria (Romero et al., 2015) and over one third will not demonstrate histological changes (Smulian et al., 1999). Subclinical histological chorioamnionitis also exists and may also infer an increased risk of morbidity (Tita and Andrews, 2010). Although it is important to note that infants defined in this way do not demonstrate signs of impaired white matter development, further investigation with histologically defined chorioamnionitis is warranted. Indeed, in a recent study using a histological diagnosis of chorioamnionitis lower FA was observed throughout the white matter. It should be noted that over three-quarters of this cohort also displayed histological evidence of fetal inflammatory response syndrome (Anblagan et al., 2016).

In this study we identified evidence of diffuse white matter abnormality associated with fetal growth restriction. Imaging studies in term neonates with fetal growth restriction demonstrate altered brain structure with reduced cortical gray matter (Tolsa et al., 2004), and hippocampal volume (Lodygensky et al., 2008), delayed cortical development (Dubois et al., 2008) and reduced FA in the corpus callosum (Padilla et al., 2014) when compared to appropriately grown infants. In a recent systematic review, fetal growth restriction was associated with a risk of poorer neurodevelopmental performance between 6 months and 3 years (Levine et al., 2015) and animal models of fetal growth restriction demonstrate delayed oligodendrocyte maturation and myelination (Tolcos et al., 2011). Fetal growth restriction can result in both a degree of hypoxia and poor nutritional delivery to affected fetuses, which may contribute to sensitizing brain tissue and increasing its susceptibility to brain injury (Van Steenwinckel et al., 2014).

A number of studies have shown that bronchopulmonary dysplasia following preterm birth is associated with worse neurodevelopmental outcome (Asztalos et al., 2016, Short et al., 2003, Synnes et al., 2016), and brain development in these infants is impaired (Boardman et al., 2007, Neubauer et al., 2015). We have previously shown that the number of days requiring mechanical ventilation is related to diffuse white matter injury independent of the effects of prematurity (Anjari et al., 2007, Ball et al., 2010). Here, we confirm those findings in a much larger sample.

The importance of early nutrition for brain growth, maturation and neurodevelopmental outcome is increasingly being appreciated. Nutritional interventions may reduce the pathogenic micro-organisms in the gut (Kapiki et al., 2007), adjust the immunological balance (Martin and Walker, 2008) and alter the gut-immune brain axis (Al-Asmakh et al., 2012). Suboptimal early nutrition is associated with impaired cognitive performance (Lucas et al., 1998) and interventions to provide both enteral and parenteral supplementation to exceed recommended macronutrient requirements are related to increased brain volumes (Tan et al., 2008). Our study demonstrates that longer duration of parenteral nutrition and, therefore, less enteral nutrition, is associated with diffuse white matter abnormality.

In our current study we observed reduced FA and elevated RD in infants with necrotizing enterocolitis who had undergone surgery, in the absence of major focal lesions on MRI. While this relationship did not persist in the multivariate analysis, only seven infants underwent surgery for necrotizing enterocolitis in this cohort. Infants with necrotizing enterocolitis have a high risk of focal brain injury and white matter atrophy on conventional MRI (Shah et al., 2008). Heterogeneity in study design, illness characteristics and follow up, along with small sample sizes has led to conflicting results when determining neurodevelopmental outcome following necrotizing enterocolitis. Meta-analysis, however, shows that necrotizing enterocolitis stage II or higher is associated with an increased risk of impairment in preterm infants, which is increased if surgical management is required (Schulzke et al., 2007).

We did not find an association between patent ductus arteriosus and white matter injury. Two recent studies have found patent ductus arteriosus treatment to be associated with worse neurodevelopmental outcome (Bourgoin et al., 2016, Janz-Robinson et al., 2015). However, neither study corrected for respiratory morbidity despite the patent ductus arteriosus groups requiring greater respiratory support. It is therefore difficult to ascertain if haemodynamically significant patent ductus arteriosus is in itself associated with impaired neurodevelopmental outcome, or with general illness severity.

Lower FA and higher RD values at term equivalent age were associated with impaired neurodevelopmental performance in a sub-group of these infants, highlighting not only that the altered dMRI measures observed in this study are consistent with previous smaller studies in preterm infants (Counsell et al., 2008, Van Kooij et al., 2012), but also that lower FA and higher diffusivity measures associated with perinatal clinical risk factors are clinically significant.

In summary, FA values were reduced and RD values were elevated in white matter in preterm infants at term equivalent age following exposure to a number of clinical risk factors. Several perinatal risk factors have an independent association with diffuse white matter injury and exposure to multiple risk factors appears to exacerbate white matter injury, supporting the multiple hit hypothesis for the variation in brain development observed following preterm birth. FA measurements may provide a biomarker for studies exploring mechanisms of white matter injury and may expedite the assessment of efficacy of early interventions in this high-risk group of infants.

The following are the supplementary data related to this article.

Supplementary tables Image 1

**Fig. s1:**
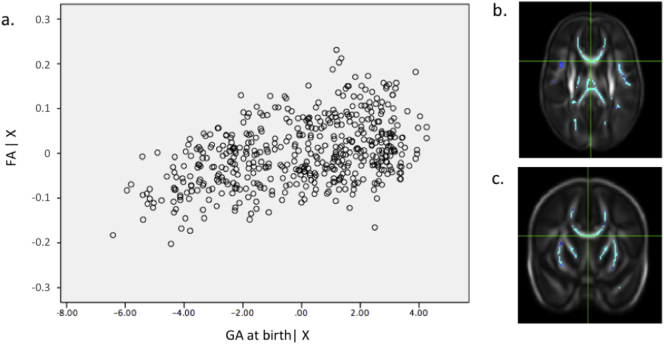
(a) Partial regression plot showing the relationship between FA values and gestational age at birth in data extracted from the most significant voxel (*r* = 0.44) highlighted in the crosshairs in the axial (b) and coronal (c) plane. PMA at scan and gender were included as covariates in the model. Key: FA | X = residuals of FA given the model; GA at birth | X = residuals of gestational age at birth given the model.

**Fig. s2:**
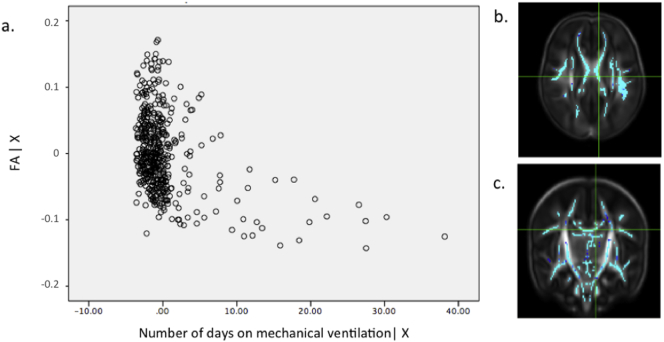
(a) Partial regression plot showing the relationship between FA values and number of days requiring mechanical ventilation in data extracted from the most significant voxel (*r* = 0.398) highlighted in the crosshairs in the axial (b) and coronal (c) plane. GA at birth, PMA at scan and gender were included as covariates in the model. Key: FA | X = residuals of FA given the model; Number of days on mechanical ventilation | X = residuals of number of days on mechanical ventilation given the model.

**Fig. s3:**
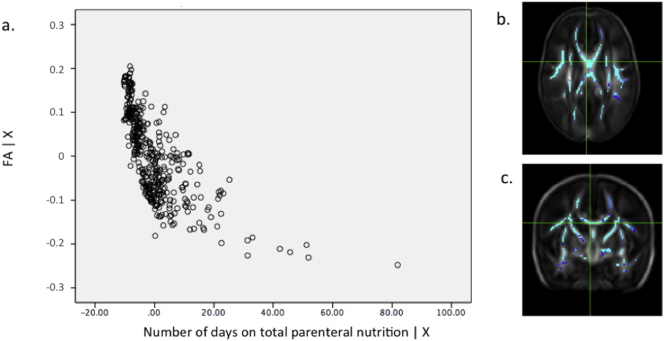
(a) Partial regression plot showing the relationship between FA values and number of days requiring parenteral nutrition in data extracted from the most significant voxel (*r* = 0.748) highlighted in the crosshairs in the axial (b) and coronal (c) plane. GA at birth, PMA at scan and gender were included as covariates in the model. Key: FA | X = residuals of FA given the model; Number of days on total parenteral nutrition | X = residuals of days on total parenteral nutrition given the model.

**Fig. s4:**
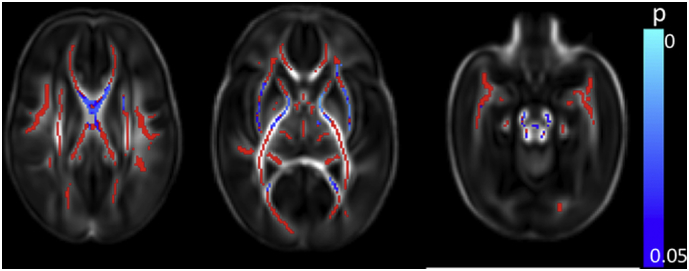
Results of multivariate analysis demonstrating lower FA values in the white matter in infants with fetal growth restriction (necrotizing enterocolitis requiring surgery, days requiring invasive ventilatory support, days requiring parenteral nutrition, GA at birth, PMA at scan and gender were included as covariates in the model). Mean FA skeleton (red) overlaid on mean FA map in axial plane. Voxels showing significant lower (p < 0.05) FA values in the white matter in infants with fetal growth restriction are shown in blue-light blue.

**Fig. s5:**
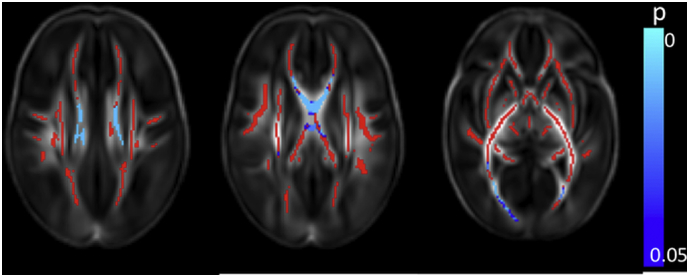
Results of multivariate analysis demonstrating a significant correlation between days requiring invasive ventilation and FA values in white matter (fetal growth restriction, necrotizing enterocolitis requiring surgery, days requiring parenteral nutrition, GA at birth, PMA at scan and gender were included as covariates in the model). Mean FA skeleton (red) overlaid on mean FA map in axial plane. Voxels showing a significant correlation (p < 0.05) between days of ventilation and FA are shown in blue-light blue.

**Fig. s6:**
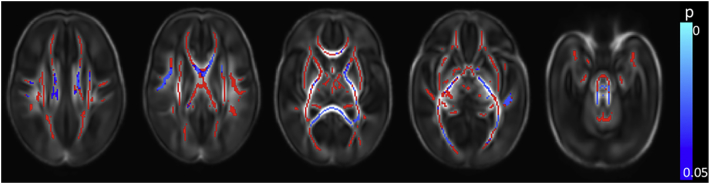
Results of multivariate analysis demonstrating a significant correlation between days requiring parenteral nutrition and dMRI measures in white matter (fetal growth restriction, necrotizing enterocolitis requiring surgery, days requiring invasive ventilatory support, GA at birth, PMA at scan and gender were included as covariates in the model). Mean FA skeleton (red) overlaid on mean FA map in the axial plane. Voxels showing a significant correlation (p < 0.05) between FA and days of parenteral nutrition with are shown in blue-light blue.

**Fig. s7:**
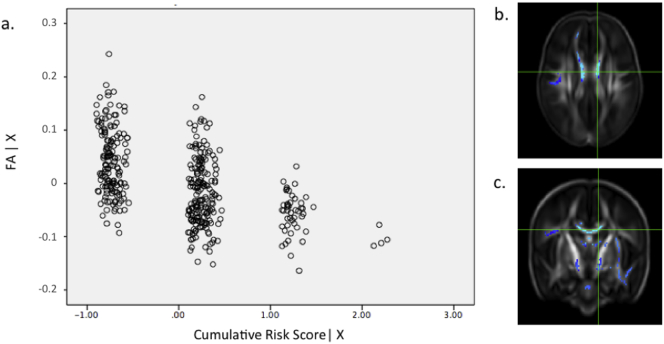
(a) Partial regression plot showing the relationship between FA values and cumulative risk score in data extracted from the most significant voxel (*r* = 0.558) highlighted in the crosshairs in the axial (b) and coronal (c) plane. GA at birth, PMA at scan and gender were included as covariates in the model. Key: FA | X = residuals of FA given the model; Cumulative risk score | X = residuals of cumulative risk score given the model.

## Conflict of interest disclosures

None reported.

## Funding/support

The work summarizes independent research supported by the National Institute for Health Research (NIHR) under its Programme Grants for Applied Research Programme (Grant Reference Number RP-PG-0707-10154) and the Department of Health via the NIHR Comprehensive Biomedical Research Centre award to Guy's and St Thomas' NHS Foundation Trust in partnership with King's College London and King's College Hospital NHS Foundation Trust. It was supported by the Medical Research Council (UK) (MR/K006355/1 and MR/L011530/1), and PhD studentship to MB. The views expressed are those of the authors and not necessarily those of the NHS, the NIHR or the Department of Health.

## References

[bb0005] Ådén U., Favrais G., Plaisant F., Winerdal M., Felderhoff-Mueser U., Lampa J., Lelièvre V., Gressens P. (2010). Systemic inflammation sensitizes the neonatal brain to excitotoxicity through a pro-/anti-inflammatory imbalance: key role of TNFalpha pathway and protection by etanercept. Brain Behav. Immun..

[bb0010] Ajayi-Obe M., Saeed N., Cowan F.M., Rutherford M.A., Edwards A.D. (2000). Reduced development of cerebral cortex in extremely preterm infants. Lancet.

[bb0015] Al-Asmakh M., Anuar F., Zadjali F., Rafter J., Pettersson S. (2012). Gut microbial communities modulating brain development and function. Gut Microbes.

[bb0020] Allen J.S., Damasio H., Grabowski T.J., Bruss J., Zhang W. (2003). Sexual dimorphism and asymmetries in the gray-white composition of the human cerebrum. NeuroImage.

[bb0025] Anblagan D., Pataky R., Evans M.J., Telford E.J., Serag A., Sparrow S., Piyasena C., Semple S.I., Wilkinson A.G., Bastin M.E., Boardman J.P. (2016). Association between preterm brain injury and exposure to chorioamnionitis during fetal life. Sci. Rep..

[bb0030] Anjari M., Srinivasan L., Allsop J.M., Hajnal J.V., Rutherford M.A., Edwards A.D., Counsell S.J. (2007). Diffusion tensor imaging with tract-based spatial statistics reveals local white matter abnormalities in preterm infants. NeuroImage.

[bb0035] Asztalos E., Church P., Riley P., Fajardo C., Shah P. (2016). Neonatal factors associated with a good neurodevelopmental outcome in very preterm infants. Am. J. Perinatol..

[bb0040] Bach M., Laun F.B., Leemans A., Tax C.M.W., Biessels G.J., Stieltjes B., Maier-Hein K.H. (2014). Methodological considerations on tract-based spatial statistics (TBSS). NeuroImage.

[bb0045] Ball G., Counsell S.J., Anjari M., Merchant N., Arichi T., Doria V., Rutherford M.a., Edwards A.D., Rueckert D., Boardman J.P. (2010). An optimised tract-based spatial statistics protocol for neonates: applications to prematurity and chronic lung disease. NeuroImage.

[bb0050] Ball G., Boardman J.P., Rueckert D., Aljabar P., Arichi T., Merchant N., Gousias I.S., Edwards A.D., Counsell S.J. (2012). The effect of preterm birth on thalamic and cortical development. Cereb. Cortex.

[bb0055] Ball G., Boardman J.P., Aljabar P., Pandit A., Arichi T., Merchant N., Rueckert D., Edwards A.D., Counsell S.J. (2013). The influence of preterm birth on the developing thalamocortical connectome. Cortex.

[bb0060] Ball G., Srinivasan L., Aljabar P., Counsell S.J., Durighel G., Hajnal J.V., Rutherford M.A., Edwards A.D. (2013). Development of cortical microstructure in the preterm human brain. Proc. Natl. Acad. Sci. U. S. A..

[bb0065] Ball G., Pazderova L., Chew A., Tusor N., Merchant N., Arichi T., Allsop J.M., Cowan F.M., Edwards A.D., Counsell S.J. (2015). Thalamocortical connectivity predicts cognition in children born preterm. Cereb. Cortex.

[bb0070] Bayley N. (2006). Bayley Scales of Infant Development.

[bb0075] Beaulieu C. (2002). The basis of anisotropic water diffusion in the nervous system - a technical review. NMR Biomed..

[bb0080] Beauport L., Schneider J., Faouzi M., Hagmann P., Huppi P.S., Tolsa J.F., Truttmann A.C., Fischer Fumeaux C.J. (2017). Impact of early nutritional intake on preterm brain: a magnetic resonance imaging study. J. Pediatr..

[bb0085] Boardman J.P., Counsell S.J., Rueckert D., Kapellou O., Bhatia K.K., Aljabar P., Hajnal J., Allsop J.M., Rutherford M.A., Edwards A.D. (2006). Abnormal deep grey matter development following preterm birth detected using deformation-based morphometry. NeuroImage.

[bb0090] Boardman J.P., Counsell S.J., Rueckert D., Hajnal J.V., Bhatia K.K., Srinivasan L., Kapellou O., Aljabar P., Dyet L.E., Rutherford M.A., Allsop J.M., Edwards A.D. (2007). Early growth in brain volume is preserved in the majority of preterm infants. Ann. Neurol..

[bb0095] Bourgoin L., Cipierre C., Hauet Q., Basset H., Gournay V., Rozé J.C., Flamant C., Gascoin G. (2016). Neurodevelopmental outcome at 2 years of age according to patent ductus arteriosus management in very preterm infants. Neonatology.

[bb0100] Brummelte S., Grunnau R.E., Chau V., Poskitt K.J., Brant R., Vinall J., Gover A., Synnes A.R., Miller S.P. (2012). Procedural pain and brain development in premature newborns. Ann. Neurol..

[bb0105] Campbell L.R., Pang Y., Ojeda N.B., Zheng B., Rhodes P.G., Alexander B.T. (2012). Intracerebral lipopolysaccharide induces neuroinflammatory change and augmented brain injury in growth-restricted neonatal rats. Pediatr. Res..

[bb0110] Chau V., Poskitt K.J., McFadden D.E., Bowen-Roberts T., Synnes A., Brant R., Sargent M.A., Soulikias W., Miller S.P. (2009). Effect of chorioamnionitis on brain development and injury in premature newborns. Ann. Neurol..

[bb0115] Chau V., Brant R., Poskitt K.J., Tam E.W., Synnes A., Miller S.P. (2012). Postnatal infection is associated with widespread abnormalities of brain development in premature newborns. Pediatr. Res..

[bb0120] Chau V., Synnes A., Grunau R.E., Poskitt K.J., Brant R., Miller S.P. (2013). Abnormal brain maturation in preterm neonates associated with adverse developmental outcomes. Neurology.

[bb0125] Connell T.G., Rele M., Cowley D., Buttery J.P., Curtis N. (2007). How reliable is a negative blood culture result? Volume of blood submitted for culture in routine practice in a children's hospital. Pediatrics.

[bb0130] Coumans A.B.C., Middelanis J., Garnier Y., Vaihinger H.M., Leib S.L., Von Duering M.U., Hasaart T.H.M., Jensen A., Berger R. (2003). Intracisternal application of endotoxin enhances the susceptibility to subsequent hypoxic-ischemic brain damage in neonatal rats. Pediatr. Res..

[bb0135] Counsell S.J., Shen Y., Boardman J.P., Larkman D.J., Kapellou O., Ward P., Allsop J.M., Cowan F.M., Hajnal J.V., Edwards A.D., Rutherford M.A. (2006). Axial and radial diffusivity in preterm infants who have diffuse white matter changes on magnetic resonance imaging at term-equivalent age. Pediatrics.

[bb0140] Counsell S.J., Edwards A.D., Chew A.T.M., Anjari M., Dyet L.E., Srinivasan L., Boardman J.P., Allsop J.M., Hajnal J.V., Rutherford M.A., Cowan F.M. (2008). Specific relations between neurodevelopmental abilities and white matter microstructure in children born preterm. Brain.

[bb0145] Dammann O., Leviton A. (2004). Inflammatory brain damage in preterm newborns - dry numbers, wet lab, and causal inferences. Early Hum. Dev..

[bb0150] Dubois J., Benders M., Borradori-Tolsa C., Cachia A., Lazeyras F., Ha-Vinh Leuchter R., Sizonenko S.V., Warfield S.K., Mangin J.F., Huppi P.S. (2008). Primary cortical folding in the human newborn: an early marker of later functional development. Brain.

[bb0155] Duerden E.G., Foong J., Chau V., Branson H., Poskitt K.J., Grunau R.E., Synnes A., Zwicker J.G., Miller S.P. (2015). Tract-based spatial statistics in preterm neonates predicts cognitive and motor outcomes at 18 months. AJNR Am. J. Neuroradiol..

[bb0160] Eklind S., Mallard C., Leverin A.L., Gilland E., Blomgren K., Mattsby-Baltzer I., Hagberg H. (2001). Bacterial endotoxin sensitizes the immature brain to hypoxic-ischaemic injury. Eur. J. Neurosci..

[bb0165] Guo T., Duerden E.G., Adams E., Chau V., Branson H.M., Chakravarty M.M., Poskitt K.J., Synnes A., Grunau R.E., Miller S.P. (2017). Quantitative assessment of white matte rinjury in preterm neonates: association with outcomes. Neurology.

[bb0170] Hüppi P.S., Maier S.E., Peled S., Zientara G.P., Barnes P.D., Jolesz F.A., Volpe J.J. (1998). Microstructural development of human newborn cerebral white matter assessed in vivo by diffusion tensor magnetic resonance imaging. Pediatr. Res..

[bb0175] Janz-Robinson E.M., Badawi N., Walker K., Bajuk B., Abdel-Latif M.E. (2015). Neurodevelopmental outcomes of premature infants treated for patent ductus arteriosus: a population-based cohort study. J. Pediatr..

[bb0180] Jarvis S., Glinianaia S.V., Arnaud C., Fauconnier J., Johnson A., McManus V., Topp M., Uvebrant P., Cans C., Krägeloh-Mann I. (2005). Case gender and severity in cerebral palsy varies with intrauterine growth. Arch. Dis. Child..

[bb0185] Jeurissen B., Leemans A., Tournier J.D., Jones D.K., Sijbers J. (2013). Investigating the prevalence of complex fiber configurations in white matter tissue with diffusion magnetic resonance imaging. Hum. Brain Mapp..

[bb0190] Kapiki A., Costalos C., Oikonomidou C., Triantafyllidou A., Loukatou E., Pertrohilou V. (2007). The effect of a fructo-oligosaccharide supplemented formula on gut flora of preterm infants. Early Hum. Dev..

[bb0195] Kersbergen K.J., Makropoulos A., Aljabar A., Groenendaal F., de Vries L.S., Counsell S.J., Benders M.J. (2016). Longitudinal regional brain development and clinical risk factors in extremely preterm infants. J. Pediatr..

[bb0200] Kuypers E., Ophelders D., Jellema R.K., Kunzmann S., Gavilanes A.W., Kramer B.W. (2012). White matter injury following fetal inflammatory response syndrome induced by chorioamnionitis and fetal sepsis: lessons from experimental ovine models. Early Hum. Dev..

[bb0205] Levine T. a, Grunau R.E., McAuliffe F.M., Pinnamaneni R., Foran A., Alderdice F. a (2015). Early childhood neurodevelopment after intrauterine growth restriction: a systematic review. Pediatrics.

[bb0210] Lodygensky G.A., Seghier M.L., Warfield S.K., Tolsa C.B., Sizonenko S., Lazeyras F., Hüppi P.S. (2008). Intrauterine growth restriction affects the preterm infant's hippocampus. Pediatr. Res..

[bb0215] Lucas A., Morley R., Cole T.J. (1998). Randomised trial of early diet in preterm babies and later intelligence quotient. BMJ.

[bb0220] Luders E., Narr K.L., Thompson P.M., Rex D.E., Woods R.P., DeLuca H., Jancke L., Toga A.W. (2006). Gender effects on cortical thickness and the influence of scaling. Hum. Brain Mapp..

[bb0225] Martin C.R., Walker W.A. (2008). Probiotics: role in pathophysiology and prevention in necrotizing enterocolitis. Semin. Perinatol..

[bb0230] Moore T., Hennessy E.M., Myles J., Johnson S.J., Draper E.S., Costeloe K.L., Marlow N. (2012). Neurological and developmental outcome in extremely preterm children born in England in 1995 and 2006: the EPICure studies. BMJ.

[bb0235] Neubauer V., Junker D., Griesmaier E., Schocke M., Kiechl-Kohlendorfer U. (2015). Bronchopulmonary dysplasia is associated with delayed structural brain maturation in preterm infants. Neonatology.

[bb0240] Padilla N., Junqué C., Figueras F., Sanz-Cortes M., Bargalló N., Arranz A., Donaire A., Figueras J., Gratacos E. (2014). Differential vulnerability of gray matter and white matter to intrauterine growth restriction in preterm infants at 12 months corrected age. Brain Res..

[bb0245] Penn A.A., Gressens P., Fleiss B., Back S.A., Gallo V. (2016). Controversies in preterm brain injury. Neurobiol. Dis..

[bb0250] Romero R., Miranda J., Kusanovic J.P., Chaiworapongsa T., Chaemsaithong P., Martinez A., Gotsch F., Dong Z., Ahmed A.I., Shaman M., Lannaman K., Yoon B.H., Hassan S.S., Kim C.J., Korzeniewski S.J., Yeo L., Kim Y.M. (2015). Clinical chorioamnionitis at term I: microbiology of the amniotic cavity using cultivation and molecular techniques. J. Perinat. Med..

[bb0255] Rose S.E., Hatzigeorgiou X., Strudwick M.W., Durbridge G., Davies P.S.W., Colditz P.B. (2008). Altered white matter diffusion anisotropy in normal and preterm infants at term-equivalent age. Magn. Reson. Med..

[bb0260] Rose J., Butler E.E., Lamont L.E., Barnes P.D., Atlas S.W., Stevenson D.K. (2009). Neonatal brain structure on MRI and diffusion tensor imaging, sex, and neurodevelopment in very-low-birthweight preterm children. Dev. Med. Child Neurol..

[bb0265] Rose J., Vassar R., Cahill-Rowley K., Guzman X.S., Stevenson D.K., Barnea-Goraly N. (2014). Brain microstructural development at near-term age in very-low-birth-weight preterm infants: an atlas-based diffusion imaging study. NeuroImage.

[bb0270] Sakuma H., Nomura Y., Takeda K., Tagami T., Nakagawa T., Tamagawa Y., Ishii Y., Tsukamoto T. (1991). Adult and neonatal human brain: diffusional anisotropy and myelination with diffusion-weighted MR imaging. Radiology.

[bb0275] Schulzke S.M., Deshpande G.C., Patole S.K. (2007). Neurodevelopmental outcomes of very low-birth-weight infants with necrotizing enterocolitis: a systematic review of observational studies. Arch. Pediatr. Adolesc. Med..

[bb0280] Segal G.S., Chamberlain J.M. (2000). Resource utilization and contaminated blood cultures in children at risk for occult bacteremia. Arch. Pediatr. Adolesc. Med..

[bb0285] Shah D.K., Doyle L.W., Anderson P.J., Bear M., Daley A.J., Hunt R.W., Inder T.E. (2008). Adverse neurodevelopment in preterm infants with postnatal sepsis or necrotizing enterocolitis is mediated by white matter abnormalities on magnetic resonance imaging at term. J. Pediatr..

[bb0290] Short E.J., Klein N.K., Lewis B.A., Fulton S., Eisengart S., Kercsmar C., Baley J., Singer L.T. (2003). Cognitive and academic consequences of bronchopulmonary dysplasia and very low birth weight: 8-year-old outcomes. Pediatrics.

[bb0295] Smith S.M., Jenkinson M., Johansen-Berg H., Rueckert D., Nichols T.E., Mackay C.E., Watkins K.E., Ciccarelli O., Cader M.Z., Matthews P.M., Behrens T.E.J. (2006). Tract-based spatial statistics: voxelwise analysis of multi-subject diffusion data. NeuroImage.

[bb0300] Smith G.C., Gutivich J., Smyser C., Pineda R., Newnham C., Tjoeng T.H., Vavasseur C., Wallendorrf M., Neil J., Inder T.E. (2011). Neonatal intensive care unit stress is associated with brain development in preterm infants. Ann. Neurol..

[bb0305] Smulian J.C., Shen-Schwarz S., Vintzileos A.M., Lake M.F., Ananth C.V. (1999). Clinical chorioamnionitis and histologic placental inflammation. Obstet. Gynecol..

[bb0310] Synnes A., Luu T.M., Moddemann D., Church P., Lee D., Vincer M., Ballantyne M., Majnemer A., Creighton D., Yang J., Sauve R., Saigal S., Shah P., Lee S.K. (2016). Determinants of developmental outcomes in a very preterm Canadian cohort. Arch. Dis. Child Fetal Neonatal Ed..

[bb0315] Takagi T., Nakamura M., Yamad M., Hikishima K., Momoshima S., Fujiyoshi K., Shibata S., Okano H. (2009). Visualisation of peripheral nerve degeneration and regeneration: monitoring with diffusion tensor tractography. NeuroImage.

[bb0320] Tan M., Abernethy L., Cooke R. (2008). Improving head growth in preterm infants—a randomised controlled trial II: MRI and developmental outcomes in the first year. Arch. Dis. Child. Fetal Neonatal Ed..

[bb0325] Thompson D.K., Lee K.J., Egan G.F., Warfield S.K., Doyle L.W., Anderson P.J., Inder T.E. (2014). Regional white matter microstructure in very preterm ibfants: predictors and 7 year outcomes. Cortex.

[bb0330] Tita A.T.N., Andrews W.W. (2010). Diagnosis and management of clinical chorioamnionitis. Clin. Perinatol..

[bb0335] Tolcos M., Bateman E., O'Dowd R., Markwick R., Vrijsen K., Rehn A., Rees S. (2011). Intrauterine growth restriction affects the maturation of myelin. Exp. Neurol..

[bb0340] Tolsa C.B., Zimine S., Warfield S.K., Freschi M., Rossignol A.S., Lazeyras F., Hanquinet S., Pfizenmaier M., Hüppi P.S. (2004). Early alteration of structural and functional brain development in premature infants born with intrauterine growth restriction. Pediatr. Res..

[bb0345] Van Kooij B.J.M., De Vries L.S., Ball G., Van Haastert I.C., Benders M.J.N.L., Groenendaal F., Counsell S.J. (2012). Neonatal tract-based spatial statistics findings and outcome in preterm infants. Am. J. Neuroradiol..

[bb0350] Van Steenwinckel J., Schang A.-L., Sigaut S., Chhor V., Degos V., Hagberg H., Baud O., Fleiss B., Gressens P. (2014). Brain damage of the preterm infant: new insights into the role of inflammation. Biochem. Soc. Trans..

[bb0355] Volanakis J. (2001). Human C-reactive protein: expression, structure, and function. Mol. Immunol..

[bb0360] Volpe J.J. (2009). Brain injury in premature infants: a complex amalgam of destructive and developmental disturbances. Lancet Neurol..

[bb0365] Wheeler-Kingshott C.A., Cercignani M. (2009). About “axial” and “radial” diffusivities. Magn. Reson. Med..

[bb0370] WHO (2013). Preterm birth [WWW Document]. Fact sheet No. 363. http://www.who.int/mediacentre/factsheets/fs363/en.

[bb0375] Wimberger D.M., Roberts T.P., Barkovich A.J., Prayer L.M., Moseley M.E., Kucharczyk J. (1995). Identification of “premyelination” by diffusion-weighted MRI. J. Comput. Assist. Tomogr..

[bb0380] Yakolev P.I., Lecours A.R., Minowski A. (1967). The myelogenic cycles of regional maturation of the brain. Regional Development of the Brain in Early Life.

[bb0385] Yang L., Sameshima H., Ikeda T., Ikenoue T. (2004). Lipopolysaccharide administration enhances hypoxic-ischemic brain damage in newborn rats. J. Obstet. Gynaecol. Res..

[bb0390] Zhang H., Yushkevich P.A., Alexander D.C., Gee J.C. (2006). Deformable registration of diffusion tensor MR images with explicit orientation optimization. Med. Image Anal..

[bb0395] Zwicker J.G., Grunau R.E., Adams E., Chau V., Bryant R., Poskitt K.J., Synnes A., Miller S.P. (2013). Score for neonatal acute physiology II and neonatal pain predict corticospinal tract development in premature newborns. Pediatr. Neurol..

