# Supplementary material for: Exploring the multiple-hit hypothesis of preterm white matter damage using diffusion MRI
Source: Neuroimage Clin. 2017 Nov 21;17:596–606. doi: 10.1016/j.nicl.2017.11.017 (PMC5716951; doi:10.1016/j.nicl.2017.11.017)
Supplement: Supplementary file 1 — Supplementary tables [file mmc1.docx]

**Supplementary Data**

Table s1. Focal brain lesions leading to exclusion from subsequent analysis

| **Lesions** | **No (%)** |
| --- | --- |
| Periventricular leukomalacia | 23 (3.7) |
| Periventricular leukomalacia with cerebellar hemorrhage / atrophy | 1 (0.2) |
| Hemorrhagic parenchymal infarction | 25 (4.1) |
| Hemorrhagic parenchymal infarction with cerebellar hemorrhage / atrophy | 3 (0.5) |
| Multiple cerebellar hemorrhages or atrophy | 13 (2.1) |
| Thalamic / basal ganglia lesions | 3 (0.5) |
| Middle cerebral artery (MCA) infarct | 1 (0.2) |
| Other cystic lesion | 2 (0.3) |
| Other hemorrhagic lesion | 4 (0.7) |

Table s2. Comparison between infants included in multivariate risk score analysis and those who were excluded due to missing data.

| **Perinatal Characteristic** | **Included in multivariate risk score analysis**  **(n = 381)** | **Excluded from multivariate risk score analysis**  **(n = 110)** | **P value** |
| --- | --- | --- | --- |
| GA at birth, median (range) in weeks | 30^+1^  (23^+2^ – 33^+4^) | 30  (24 – 33^+5^) | 0.35 |
| PMA at scan, median (range) in weeks | 42^+2^  (38 – 45) | 42  (38 – 44^+5^) | 0.71 |
| Males, no (%) | 197 (51.7) | 59 (53.6) | 0.23 |
| Birth set, singletons/twins, no (%) | 272(71.4)/  109 (28.6) | 74(67.3)/  36 (32.7) | 0.18 |

Table s3. Summary of findings

| **Perinatal Risk Factor** | **FA** | **AD** | **MD** | **RD** |
| --- | --- | --- | --- | --- |
| Gestational age at birth | +ve correlation throughout the white matter | -ve correlation in ALIC, PLIC, corpus callosum, fornix, optic radiation, ILF /IFOF.  +ve correlation centrum semiovale | -ve correlation in corpus callosum, fornix, external capsule, ALIC, PLIC, optic radiation, ILF/IFOF, right crus cerebri | -ve correlation in cingulum, corpus callosum, external capsule, ALIC, PLIC, optic radiation, ILF/IFOF, crus cerebri, left frontal white matter |
| Male sex | Higher in right PLIC and right crus cerebri | Higher in centrum semiovale, PLIC, right SLF, right ALIC, right optic radiation, right crus cerebri | Higher in centrum semiovale, SLF, ALIC, PLIC, optic radiation, left frontal white matter | Higher in centrum semiovale, SLF, ALIC, optic radiation, right PLIC |
| Chorioamnionitis | No significant differences | No significant differences | No significant differences | No significant differences |
| Fetal growth restriction | Lower in corpus callosum, external capsule, ALIC, PLIC, fornix, optic radiation, ILF/IFOF, crus cerebri, cerebellar peduncles right cingulum and SLF. | No significant differences | No significant differences | No significant differences |
| Days on mechanical ventilation | -ve correlation throughout the white matter | -ve correlation in the left ALIC, left PLIC, left external capsule, left crus cerebri, left anterior ILF | No significant correlations | +ve correlation in the corpus callosum |
| Days on parenteral nutrition | -ve correlation in most white matter regions | No significant correlations | No significant correlations | No significant correlations |
| Necrotizing enterocolitis | Lower throughout the white matter | No significant differences | No significant differences | Higher in the centrum semiovale, right SLF, a small region of the right ALIC, right ILF/IFOF |
| Patent Ductus Arteriosus | No significant differences | No significant differences | No significant differences | No significant differences |
| Cumulative perinatal risk factor score | -ve correlation in the corpus callosum, fornix, optic radiation, ILF/IFOF, crus cerebri, cerebellar peduncles, left frontal white matter, left SLF, left cingulum, left ALIC, left PLIC and left external capsule. | No significant correlations | No significant correlations | +ve correlation in the corpus callosum and the left fornix |
| **Developmental performance** |  |  |  |  |
| Cognition | +ve correlation throughout the white matter | No significant correlations | No significant correlations | No significant correlations |
| Motor | +ve correlation in corpus callosum, PLIC, fornix, optic radiation, ILF /IFOF, crus cerebri, cerebellar peduncles, right ALIC, right external capsule. | No significant correlations | No significant correlations | -ve correlation in the corpus callosum, small areas in the optic radiation, left fornix |
| Language | +ve correlation in the body of the corpus callosum, left fornix, anterior aspect of right ILF/IFOF | No significant correlations | No significant correlations | No significant correlations |

**Legends to supplementary figures**

Figure s1. (a) Partial regression plot showing the relationship between FA values and gestational age at birth in data extracted from the most significant voxel (r = 0.44) highlighted in the crosshairs in the axial (b) and coronal (c) plane. PMA at scan and gender were included as covariates in the model. Key: FA | X = residuals of FA given the model; GA at birth | X = residuals of gestational age at birth given the model.

Figure s2. (a) Partial regression plot showing the relationship between FA values and number of days requiring mechanical ventilation in data extracted from the most significant voxel (r = 0.398) highlighted in the crosshairs in the axial (b) and coronal (c) plane. GA at birth, PMA at scan and gender were included as covariates in the model. Key: FA | X = residuals of FA given the model; Number of days on mechanical ventilation | X = residuals of number of days on mechanical ventilation given the model.

Figure s3. (a) Partial regression plot showing the relationship between FA values and number of days requiring parenteral nutrition in data extracted from the most significant voxel (r = 0.748) highlighted in the crosshairs in the axial (b) and coronal (c) plane. GA at birth, PMA at scan and gender were included as covariates in the model. Key: FA | X = residuals of FA given the model; Number of days on total parenteral nutrition | X = residuals of days on total parenteral nutrition given the model.

Figure s4. Results of multivariate analysis demonstrating lower FA values in the white matter in infants with fetal growth restriction (necrotizing enterocolitis requiring surgery, days requiring invasive ventilatory support, days requiring parenteral nutrition, GA at birth, PMA at scan and gender were included as covariates in the model). Mean FA skeleton (red) overlaid on mean FA map in axial plane. Voxels showing significant lower (p<0.05) FA values in the white matter in infants with fetal growth restriction are shown in blue-light blue.

Figure s5. Results of multivariate analysis demonstrating a significant correlation between days requiring invasive ventilation and FA values in white matter (fetal growth restriction, necrotizing enterocolitis requiring surgery, days requiring parenteral nutrition, GA at birth, PMA at scan and gender were included as covariates in the model). Mean FA skeleton (red) overlaid on mean FA map in axial plane. Voxels showing a significant correlation (p<0.05) between days of ventilation and FA are shown in blue-light blue.

Figure s6. Results of multivariate analysis demonstrating a significant correlation between days requiring parenteral nutrition and dMRI measures in white matter (fetal growth restriction, necrotizing enterocolitis requiring surgery, days requiring invasive ventilatory support, GA at birth, PMA at scan and gender were included as covariates in the model). Mean FA skeleton (red) overlaid on mean FA map in the axial plane. Voxels showing a significant correlation (p<0.05) between FA and days of parenteral nutrition with are shown in blue-light blue.

Figure s7. (a) Partial regression plot showing the relationship between FA values and cumulative risk score in data extracted from the most significant voxel (r = 0.558) highlighted in the crosshairs in the axial (b) and coronal (c) plane. GA at birth, PMA at scan and gender were included as covariates in the model. Key: FA | X = residuals of FA given the model; Cumulative risk score | X = residuals of cumulative risk score given the model.
